# Effect of Synthesized C-S-H Nanoparticles on the Early Hydration and Microstructure of Cement

**DOI:** 10.3390/ma18143396

**Published:** 2025-07-20

**Authors:** Yoojung Hwang, Suji Woo, Young-Cheol Choi

**Affiliations:** 1 Department of Civil and Environmental Engineering, Gachon University, Seongnam 13120, Gyeonggi-do, Republic of Korea; yoojung200311@gachon.ac.kr; 2 Construction Material Research Team, GS E&C, 33 Jong-ro, Jongno-gu, Seoul 03159, Republic of Korea

**Keywords:** C-S-H nanoparticles, X-ray diffraction, cement hydration, nucleation site, ground granulated blast-furnace slag cement

## Abstract

Ground granulated blast-furnace slag (GGBS), a waste product generated during steel production, can be added as a substitute for cement in concrete to mitigate the environmental impact of the cement and steel industries. However, the use of GGBS is limited because it decreases the early strength development of cement or concrete. This study evaluated the performance of incorporating synthesized C-S-H nanoparticles to enhance the compressive strength, early hydration, and microstructure of cement composite. The synthesized C-S-H nanoparticles were produced at standard atmospheric pressure and room temperature. Heat of hydration, X-ray diffraction, and thermogravimetric analyses were conducted to investigate the hydration and mechanical properties of the cement containing the C-S-H nanoparticles. Further, mercury intrusion porosimetry was conducted to examine the pore structures. The experimental finding demonstrated that adding C-S-H nanoparticles accelerated the early hydration progress in the cement composites, thereby increasing their initial compressive strength.

## 1. Introduction

Concrete is the most commonly used construction material in the world [1,2], and the cement in concrete is the most important component determining its properties [3]. The global production of cement exceeds four billion tons annually, rendering it the second-highest contributor to CO_2_ emissions across all industries [4,5,6]. The growing importance of environmental protection and the various global agreements on CO_2_ reduction require minimizing CO_2_ emissions from cement production. However, achieving this reduction has proven to be very challenging [7].

Numerous studies have focused on identifying cement substitutes to lower the overall cement content in concrete [8,9,10]. For instance, fly ash (FA), a pozzolanic material, and ground granulated blast-furnace slag (GGBS), a byproduct of steel production, possess latent hydraulic properties and are commonly used as cement substitutes [11,12]. These materials can partially replace cement, providing environmental benefits and enhancing the mechanical properties and durability of concrete. However, cement and concrete with a high content of FA or GGBS exhibit lower early-age strength compared to ordinary concrete due to a slower initial hydration reaction [13]. This limitation has led to various studies exploring the use of hydration accelerators or strength enhancers to mitigate this issue.

Some studies confirmed that chloride-based hydration accelerators are effective in improving the initial strength of concrete, however, they reduce the long-term strength as compared to that of ordinary concrete [14,15]. It was reported that concrete containing sodium-sulfate-based hydration accelerators can help improve the initial strength by rapidly generating ettringite(Aft) hydration products, thereby accelerating the early-age hydration of C_3_A and C_3_S [16,17]. However, sodium-sulfate-based hydration accelerators reduce the long-term strength, similar to chloride-based hydration accelerators. In addition to reducing the long-term strength of concrete, chloride- and sodium-based hydration accelerators shorten the service life of structures by accelerating long-term deterioration, such as the corrosion of steel reinforcement bars [18] and alkali–aggregate reactions [19].

Recent studies have employed calcium silicate hydrate (C-S-H) seeds as hydration accelerators, as they do not adversely affect the long-term durability of cement-based structures. C-S-H, one of the primary hydration products of Portland cement, plays a critical role in determining the mechanical properties and durability of cementitious materials. It is formed through the hydration of tricalcium silicate (C_3_S) and dicalcium silicate (C_2_S), the two major constituents of cement clinker. Although the structure of C-S-H is poorly crystalline and varies depending on hydration conditions, it forms a dense, cohesive gel that fills capillary pores and binds solid particles together. This gel-like phase is responsible for the majority of strength development in hardened cement paste and is widely regarded as the principal strength-giving phase in cement-based systems [20,21]. Therefore, understanding the morphology, composition, and long-term evolution of C-S-H is essential for the design and performance optimization of durable cementitious materials.

Building on this knowledge, recent approaches have focused on using engineered C-S-H nanocomposites to enhance early-age strength by accelerating the hydration process. These materials incorporate ultra-fine particles smaller than cement grains, which serve as nucleation sites for hydration products, thereby expediting reaction kinetics [22,23,24]. For instance, C-S-H–polycarboxylate nanocomposites with particle sizes below 50 nm were synthesized in an isoprenyloxy poly(ethylene glycol)-based superplasticizer solution by adjusting the pH during synthesis [25]. Application of these nanocomposites led to a 100% increase in the 12-h compressive strength of cement specimens. Another study fabricated a 329 nm C-S-H–polycarboxylate nanocomposite using a methallyl-ether-based polycarboxylate solution and reported a 55% strength improvement in mortar specimens at 12 h [26].

John et al. [27] investigated the influence of the Ca/Si ratio in C-S-H nanoparticles on cement hydration. Their findings indicated that lower Ca/Si ratios result in a higher specific surface area for C-S-H, thereby further accelerating cement hydration. Furthermore, it was shown that C-S-H seeds accelerated the hydration reaction of cement and enhanced its compressive strength by at least 20% [28]. As the use of C-S-H increased, the early strength development of cement composites improved [26,29]. Moreover, under low-temperature curing conditions, it has been reported that C-S-H nanoparticles can serve as accelerators to enhance compressive strength [30].

In this study, C-S-H nanoparticles were synthesized using reagent-class sodium metasilicate (Na_2_SiO_3_∙5H_2_O), cobalt salts (Ca(NO_3_)_2_∙4H_2_O), and a dispersing agent (polycarboxylate based). X-ray photoelectron spectroscopy (XPS) analysis was conducted to analyze the Ca/Si ratios of the synthesized C-S-H nanoparticles. Cement pastes were prepared by adding C-S-H nanoparticles. Hydration heat measurements, thermogravimetry (TG), mercury intrusion porosimetry (MIP), and X-ray diffraction (XRD) analyses were conducted to investigate the cement hydration properties. The hydration behavior of GGBS cement was also examined for its response to the influence of synthesized C-S-H nanoparticles. To this end, a heat of hydration test was conducted along with TG and MIP at each age. Further, the effect of the nanoparticles on the prepared mortar specimens was examined. These findings are expected to promote the wider application of GGBS addition to cement to reduce its environmental impact while ensuring sufficient mechanical properties by modification with C-S-H nanoparticles.

## 2. Materials and Methods

### 2.1. Materials

**Figure 1:**
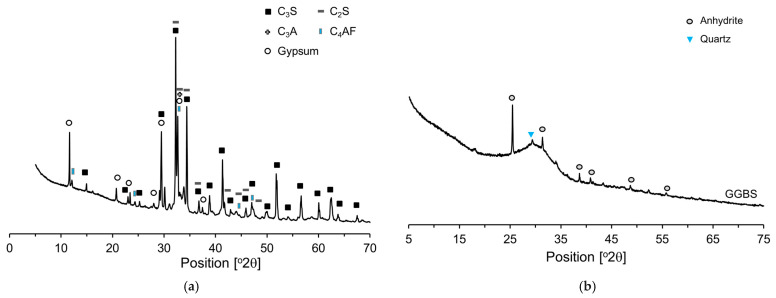
XRD patterns: (**a**) RC; (**b**) GGBS.

**Figure 2:**
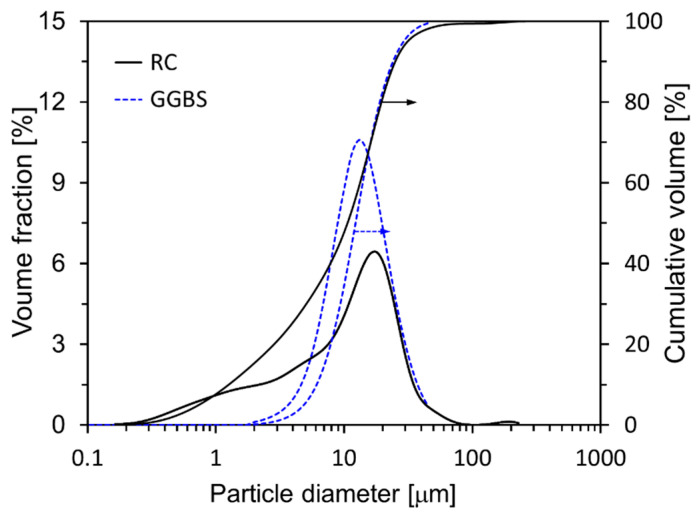
Particle size distributions of RC and GGBS.

In this research, a cement with no supplementary cementitious materials such as FA or GGBS was used as the binder, referred to as research cement (RC). RC was produced by mixing desulfurized gypsum (5%) with clinker (95%) and then crushing the mixture [29]. RC used more reliable results when analyzing the cement microstructure. Table 1 presents the chemical compositions of the raw materials and their respective loss-on-ignition (LOI) values. The main mineral components of the RC were identified by XRD (Figure 1a) as C_3_S, C_2_S, C_3_A, C_4_AF, and gypsum, and Rietveld refinement of the XRD pattern yielded fractions of 61.8, 10.6, 11.3, 8.6, and 7.7 wt%, respectively. The main mineral components of the GGBS were anhydrite and quartz. Most of the GGBS is amorphous, with small amounts of crystalline phases, namely, anhydrite (2.9%) and quartz (2.1%). The densities of RC and GGBS are 3.18 g/cm^3^ and 2.91 g/cm^3^, respectively, and their Blaine fineness values are 3870 cm^2^/g and 4200 cm^2^/g. The particle size distribution characteristics of RC and GGBS are illustrated in detail in Figure 2, providing a comprehensive depiction of the range and proportion of particle sizes present in each material. C-S-H nanoparticles, used as a hardening accelerator, were synthesized using sodium metasilicate (Na_2_SiO_3_∙5H_2_O), calcium nitrate tetrahydrate (Ca(NO_3_)_2_∙4H_2_O) powder, and a polycarboxylate based dispersant (50 wt% solid content).

### 2.2. Synthesis of C-S-H Nanoparticles

**Figure 3:**
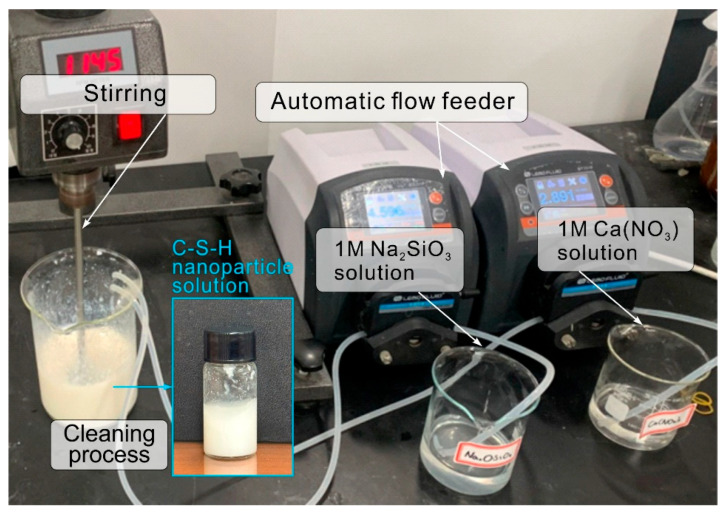
Setup for synthesizing C-S-H nanoparticles.

C-S-H nanoparticles synthesized in the authors’ previous research were used [31]. The synthesis of C-S-H nanoparticle was carried out at room temperature using reagent-class Na_2_SiO_3_∙5H_2_O and Ca(NO_3_)_2_∙4H_2_O powders. Figure 3 illustrates an overview of the C-S-H nanoparticle synthesis process. First, 60 g of distilled water was mixed with 22.34 g of the polycarboxylate superplasticizer in a beaker, and the mixture was stirred using an agitator. The content of the polycarboxylate superplasticizer was used to adjust the crystal size of the C-S-H nanoparticles [32]. The reagent-class Na_2_SiO_3_∙5H_2_O and Ca(NO_3_)_2_∙4H_2_O powders were added to 180 g and 90 g of distilled water, respectively, to achieve a molar concentration ratio of 1:1. Thereafter, automatic flow feeders were fixed to the beaker to supply rates of 4.6 and 2.9 mL/min of Na_2_SiO_3_ and Ca(NO_3_)_2_ solutions, respectively, under stirring at a speed of at least 1000 rpm. During this synthesis, Na^+^ and NO^3−^ are generated as impurities, in addition to the C-S-H nanoparticles. These components must be considered because they reduce the durability of concrete via alkali–aggregate reactions that produce NaNO_3_ [33,34]. In this study, centrifugal separation was performed at a speed of ~4500 rpm to remove the impurities generated. The supernatant containing the impurities was removed by rinsing the precipitate with distilled water. This process was repeated three times to produce C-S-H nanoparticles as an opaque white gel with a solid content of ~21% (Figure 3). In this study, the particle size of the synthesized C-S-H nanoparticles ranges from 30 nm to 50 nm.

### 2.3. Preparation of Paste and Mortar Samples

Table 2 presents the mixture proportions of specimens. The main variables are the amount of C-S-H added and whether GGBS is used. The PCSH specimens were prepared by adding 0, 1, 2, or 3 wt% of C-S-H nanoparticles, based on the weight of RC, considering the solid content in the nanoparticle solution shown in Figure 3. The GCSH specimens were prepared by adding 0 or 3 wt% C-S-H nanoparticles to cement containing 50 wt% GGBS. The last row in Table 2, labeled “SP (wt%) in the C-S-H nanoparticle solution”, refers to the SP dosage expressed as the weight percentage relative to the total mass of the C-S-H nanoparticle suspension. For all specimens, the water-to-binder (W/B) ratio was fixed at 0.5; the binder-to-sand ratio was fixed at 1:3, and standard sand (ISO 679) was used as sand. Paste specimens were prepared to analyze the effect of the C-S-H nanoparticles on the hydration reaction of cement composites, for which the same recipes summarized in Table 2 were used, but without sand. Paste specimens were prepared following the same mixture proportions outlined in Table 2, with the exception that sand was omitted from the formulation to isolate the effects of the binder and other components on the hydration and strength development processes.

### 2.4. Characterization Methods

XPS analysis was conducted to analyze the calcium-to-silicon (Ca/Si) ratio in the C-S-H nanoparticles. Samples of dried C-S-H nanoparticles were pressed into pellets, and the sample surface was subjected to qualitative and quantitative analyses. A NEXSA XPS system (ThermoFisher Scientific, Waltham, MA, USA) was used, with the X-ray spot diameter set to 400 μm.

The hydration heat measurements were carried out via a Calmtrix I-Cal 2000 (Calmetrix, Inc., Boston, MA, USA). Pastes were prepared following the mixtures shown in Table 2. Subsequently, approximately 70 g of each specimen were carefully transferred into a dedicated test container designed to prevent external contamination. The container was then placed into the calorimeter, where, under controlled conditions of 20 ± 1 °C, the heat of hydration was continuously measured for 72 h.

Mortar specimens, prepared in accordance with the specifications outlined in ISO 679 [35], were subjected to compressive strength tests. Steel molds were employed to fabricate mortar specimens measuring 40 mm × 40 mm × 160 mm. The specimens were demolded after curing in a constant temperature (20 ± 1 °C) and relative humidity (≥90%) chamber for 24 h. During the first 24 h after casting, the specimens were cured exclusively in a controlled temperature and humidity chamber, without any exposure to water. After demolding at 1 day, they were transferred to a water tank maintained at 20 ± 1 °C for subsequent curing until the designated testing age. According to ISO 679, one specimen was split into two pieces, and the compressive strength was measured for six specimens per group. The compressive strength results were reported as average values. However, the compressive strength at ages of 8 h, 16 h, and 1 d was measured without water curing; the specimens were demolded in the chamber at the respective testing ages and tested immediately.

XRD analysis (Empyrean, Malvern Panalytical, Chipping Norton, Australia) was conducted to identify the crystalline hydration products at each age. Diffraction patterns were obtained over a 2θ range of 5–75°, with a step size of 0.01° and a counting time of 5 s, respectively. Rietveld refinement of the X-ray diffraction (XRD) data was carried out using X’Pert HighScore Plus 5.3a (PANalytical, Almelo, The Netherlands). For the quantitative analysis of mineral composition, 10% corundum (α-Al_2_O_3_) was used as an internal standard. The synthesized C-S-H nanoparticles were subjected to freezing and drying preprocessing. The cement pastes were carefully submerged in isopropanol at specific ages. Then, the samples were kept under vacuum for 24 h to halt the hydration reaction, and then dried in a furnace at 40 °C for an additional 24 h. Finally, the samples were crushed into powder before testing.

TG analysis was performed using an SDT Q600 (TA Instruments, New Castle, DE, USA) to investigate the paste’s hydration behavior at different ages. The weight change was measured while the temperature was increased to 1000 °C at a rate of 10 °C/min in a nitrogen atmosphere (100 mL/min). The samples were prepared using the same method as that used for preprocessing the paste specimens for XRD.

MIP was performed using an Autopore V system (Micromeritics, Norcross, GA, USA) to measure the pore size distribution inside the paste specimens. The specimens were fabricated by drying the paste at 40 °C for 24 h after stopping hydration by crushing it into a size of ~5 mm.

## 3. Results and Discussion

### 3.1. Effect of C-S-H Nanoparticles on Hydration Properties

**Figure 4:**
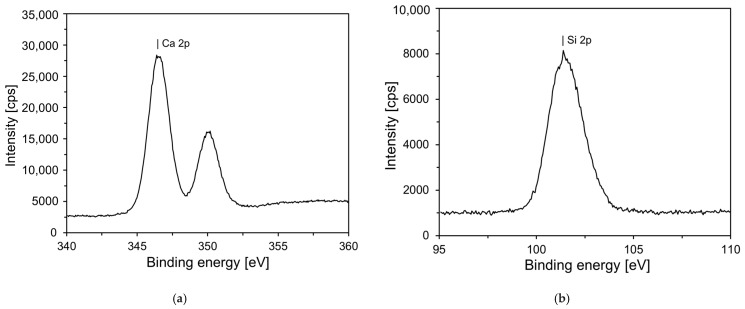
XPS analysis of C-S-H nanoparticles, showing the elemental peaks: (**a**) calcium (Ca); (**b**) silicon (Si).

Figure 4 shows the XPS results for the synthesized C-S-H nanoparticles. According to a previous study, the properties of nano C-S-H crystals vary depending on the Ca/Si ratio [36]. Figure 4a shows that the Ca 2p spectrum has two peaks at binding energies of 350.1 and 346.5 eV with intensities of 16,247 and 28,363, respectively. The Si 2p spectrum has one peak at a binding energy of 101.4 eV, with an intensity of 8141. From these results, it was determined that the Ca/Si ratio of the synthesized C-S-H nanoparticles was 0.78. A comparison of the Ca and Si peaks among the various types of C-S-H revealed that they are similar to those of 11 Å Tobermorite (Ca_4.5_(Si_6_O_16_)(OH)·5H_2_O) [34].

**Figure 5:**
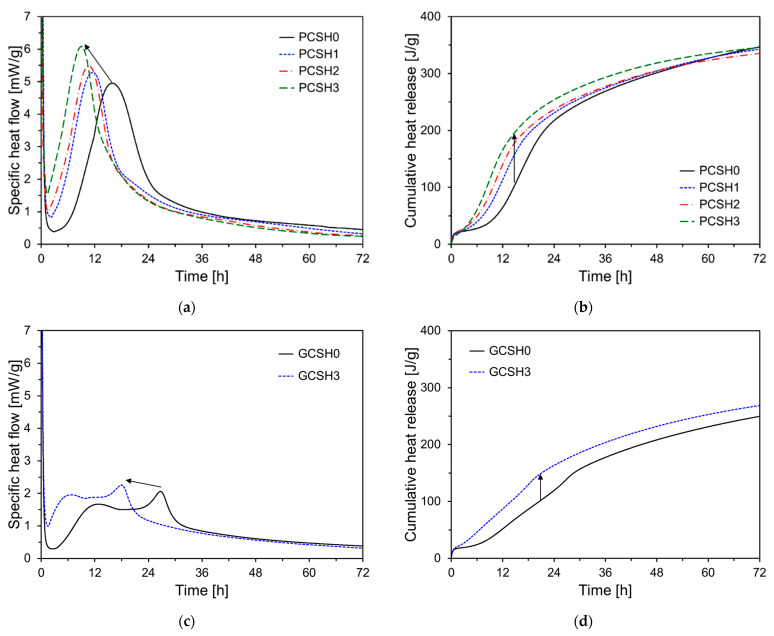
Heat of hydration results for PCSH and GCSH samples: (**a**) specific heat flow of PCSH samples; (**b**) cumulative heat release of PCSH samples; (**c**) specific heat flow of GCSH samples; (**d**) cumulative heat release of GCSH samples.

Figure 5 illustrates the specific heat flow and cumulative heat release results. Figure 5a,b show the experimental results for PCSH, and Figure 5c,d show the results for GCSH. As depicted in Figure 5a, the second peak shifted to an earlier time with increasing C-S-H nanoparticle content. This effect can be explained by the shortened induction period in the cement paste resulting from the incorporation of C-S-H nanoparticles. Furthermore, as the C-S-H content increased, the second peak grew in magnitude, indicating that C-S-H nanoparticles enhance the cement hydration reaction [37]. As shown in Figure 5b, the initial cumulative heat release of the PCSH samples increases with higher C-S-H nanoparticle content. This tendency is noticeable between 6 and 24 h and is related to the C-S-H nanoparticles accelerating the hydration reaction of cement by serving as nucleation sites [37]. However, the increment of cumulative heat release of the specimens containing the C-S-H nanoparticles decreased gradually after 24 h and was lower than that of PCSH0 at 72 h. This is attributed to the rapid and increased formation of outer C-S-H hydration products on the surface of cement particles induced by the C-S-H nanoparticles. This hydration layer hinders the contact between unreacted cement and water, thereby slowing down the hydration process.

The specific heat flow results for GCSH0 and GCSH3 are shown in Figure 5c, where the accelerated hydration reaction in GGBS cement due to the presence of C-S-H nanoparticles is observed as the earlier appearance of the second and third peaks with slightly higher intensities. At 72 h, the GCSH samples exhibited a lower cumulative heat release than the PCSH samples as shown in Figure 5d. This difference can be explained by the influence of GGBS [38]. C-S-H nanoparticles significantly enhanced the cumulative heat release in cement mixtures with a high GGBS content. This increase is likely due to the nanoparticles providing additional nucleation sites, which accelerate the hydration reaction of both the cement and the GGBS particles, thereby improving the overall reactivity of the system.

### 3.2. Effect of C-S-H Nanoparticles on the Compressive Strength

**Figure 6:**
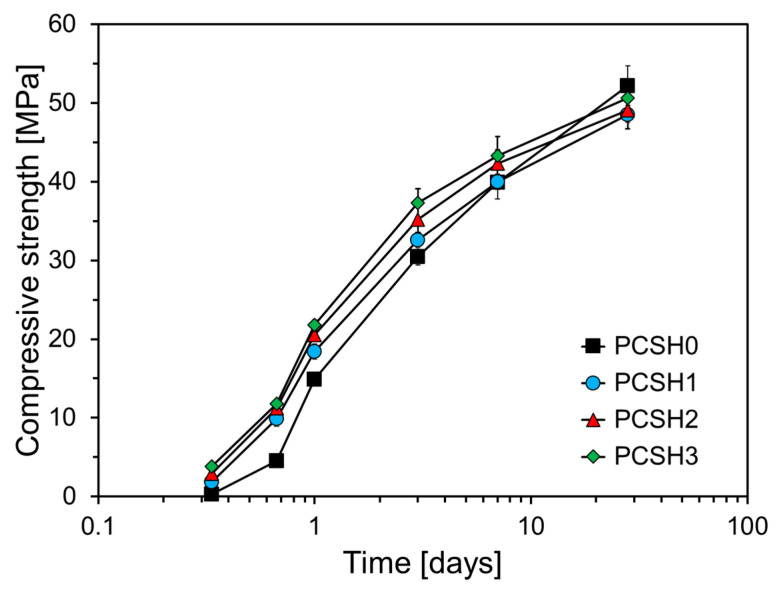
Compressive strength development over time of PCSH samples with different C-S-H nanoparticle contents.

The compressive strength tests were conducted at ages of 8 h, 16 h, 1 d, 3 d, 7 d, and 28 d. As illustrated in Figure 6, the compressive strength of mortar specimens increases with the incorporation of higher concentrations of C-S-H nanoparticles. This trend is particularly evident at early ages, though the rate of strength gain diminishes over time. All specimens containing C-S-H nanoparticles exhibited higher compressive strengths than did PCSH0 from 8 h to 7 d of age, with the largest difference observed at 16 h. At this age, the compressive strengths of PCSH1, PCSH2, and PCSH3 were 5.3, 6.6, and 7.2 MPa higher than that of PSCH0, respectively. This phenomenon can be explained by the accelerated hydration process observed in specimens with C-S-H nanoparticles, along with a reduced compressive strength gap after 16 h. By 7 d, the compressive strengths of PCSH1, PCSH2, and PCSH3 demonstrated notable improvements, exceeding the strength of PCSH0 by 0.1 MPa, 2.4 MPa, and 3.4 MPa, respectively. However, by 28 d, the compressive strengths of all specimens incorporating C-S-H nanoparticles were observed to be lower than that of PCSH0. For example, PCSH3 had the lowest compressive strength of all samples at 28 d, which was 1.6 MPa lower than that of PCSH0.

During early-age hydration, the reaction in cement samples is mainly confined to the surfaces of the cement particles [39,40]. However, C-S-H nanoparticles—smaller than the cement particles—supply extra nucleation sites that speed up the cement’s initial hydration reaction [37]. Specifically, the C-S-H nanoparticles fill the voids between cement particles and act as additional nucleation sites, facilitating the formation of hydration products through their interaction with the surrounding matrix. This increases the density of the structure compared to that of the samples without C-S-H nanoparticles, resulting in a higher initial strength up to 7 d. The hydration product layer resulting from the rapid hydration reaction reduces the diffusion rate of Ca^2+^ inside the cement particles, thereby impeding the mid-to-long-term hydration reaction [22]. This explains the significant early-age strength development observed in specimens containing C-S-H nanoparticles, however, their compressive strength beyond 28 d was relatively low.

**Figure 7:**
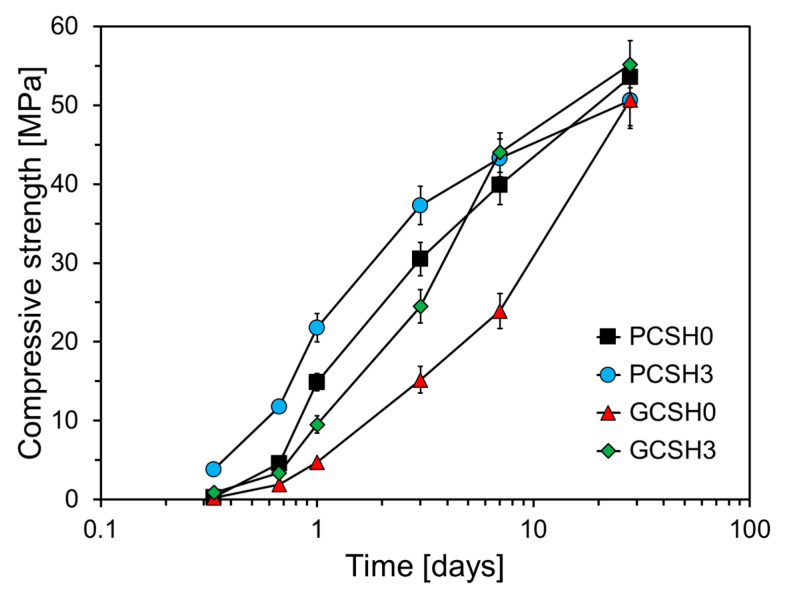
Compressive strength development over time of PCSH and GCSH samples with different C-S-H nanoparticle contents.

Figure 7 shows the compressive strength of the GGBS cement specimens containing C-S-H nanoparticles. The major limitation of cement composites containing GGBS is that their early-age strength is low compared to that of ordinary cement composites [41]. This limits the mass utilization of GGBS in the construction industry because it increases the construction time. Many studies have focused on increasing the early-age strength of cement composites containing GGBS [38,42,43,44]. As an example, Xu et al. conducted a study on the influence of nanosilica in cement composites containing high volume GGBS [38]. Figure 7 shows that the compressive strength of GCSH0, in which GGBS replaced 50% of the cement weight, is lower than that of PCSH0. The compressive strengths of GCSH0 at 1 d, 3 d, 7 d of age were 10.2, 15.3, and 16.0 MPa lower than that of PCSH0, respectively, however, the difference in compressive strength decreased to 2.9 MPa at 28 d. Furthermore, GCSH3 containing 3 wt% C-S-H nanoparticles exhibited an improved compressive strength, which was noticeable at 7 d of age. At 8 h, the compressive strength of GCSH3 surpassed that of PCSH0 by 0.57 MPa, however, it was 1.19, 5.4, and 6 MPa lower at 16 h, 1 d, and 3 d, respectively. This tendency was reversed after 3 d; at 7 and 28 d, the compressive strengths of GCSH3 were 4.1 and 1.6 MPa higher than that of PCSH0. GCSH3 consistently demonstrated higher compressive strength than GCSH0 at all ages, with the maximum difference of 20.1 MPa observed at 7 d. However, from 8 h to 3 d, the compressive strength of GCSH3 was 2.96–12.8 MPa lower than that of PCSH3. This is because the C-S-H nanoparticles act as additional nucleation sites, which accelerate the early hydration of the cement. As a result, an alkaline environment is rapidly established, which in turn activates the latent hydraulic properties of GGBS, leading to earlier and greater strength development [45].

### 3.3. Hydration Reaction Analysis by XRD

**Figure 8:**
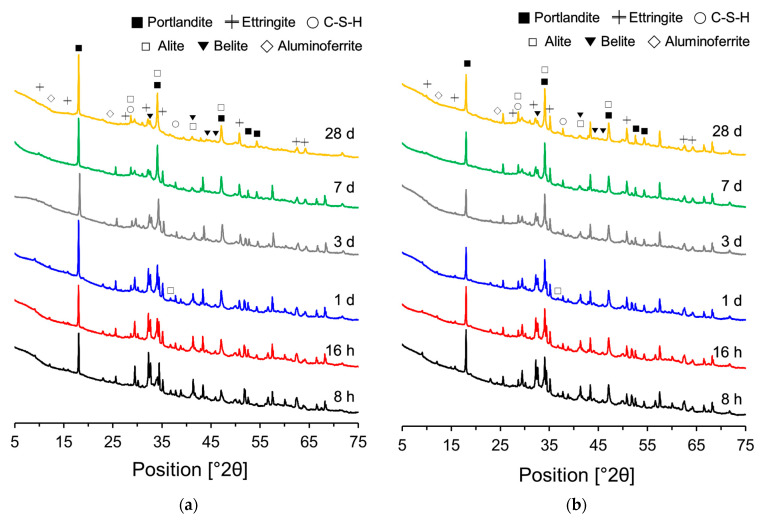
XRD patterns measured at different ages: (**a**) PCSH0; (**b**) PCSH3.

Figure 8 shows the XRD patterns of the PCSH0 and PCSH3 specimens obtained at different ages. Alite, belite, aluminoferrite, portlandite, ettringite, and C-S-H were detected in both specimens. The PCSH3 specimen had higher consumptions of alite, belite, and aluminoferrite than did PCSH0 at early-age hydration. The same tendency was observed in a previous study [29] when the results of the two samples were compared. This implies that C-S-H nanoparticles increase hydrate formation by accelerating the early-age hydration reaction [46].

**Figure 9:**
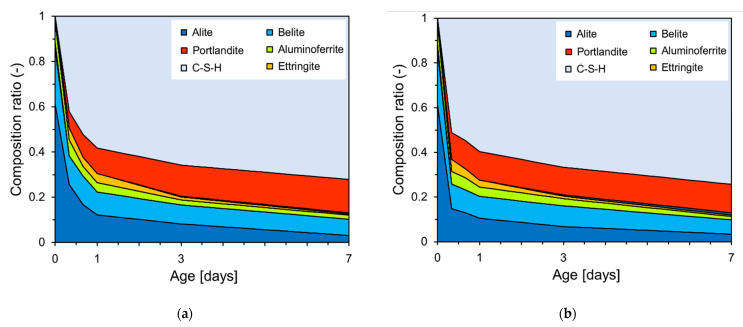
Fractions of the various crystalline components determined from Rietveld analysis of the XRD patterns: (**a**) PCSH0; (**b**) PCSH3.

Figure 9 shows the Rietveld refinement analysis results based on the XRD patterns in Figure 8. Figure 9a,b show that the generation of portlandite and C-S-H hydration products increased with age. The composition ratio (*y*-axis) was calculated by weight fraction. The quantity of C-S-H generated at the early stages of hydration was found to be approximately 9% higher in PCSH3 compared to PCSH0.

**Figure 10:**
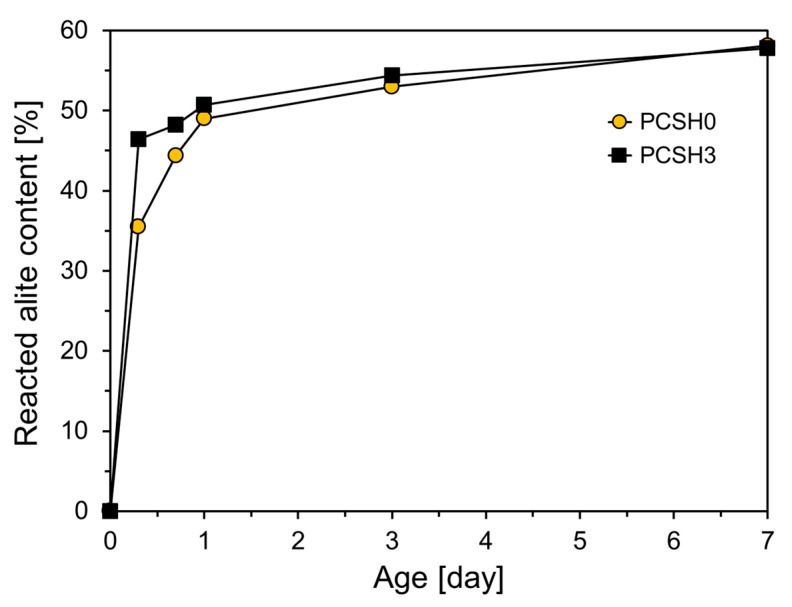
Reacted alite contents of PCSH0 and PCSH3.

The generation of C-S-H and portlandite increased with increasing consumption of alite as shown in Figure 10. The PCSH0 and PCSH3 specimens showed the most noticeable differences in alite reduction at early ages, as depicted in Figure 10. The reacted alite contents of PCSH3 were 10.9, 3.8, 1.7, and 0.4% higher than those of PCSH0 at 8 h, 16 h, 1 d, and 3 d, respectively. This is related to the finding that the PCSH3 specimen had a higher early-age compressive strength than that of PCSH0 [47].

### 3.4. Thermal Gravimetric Analysis (TGA) Results

**Figure 11:**
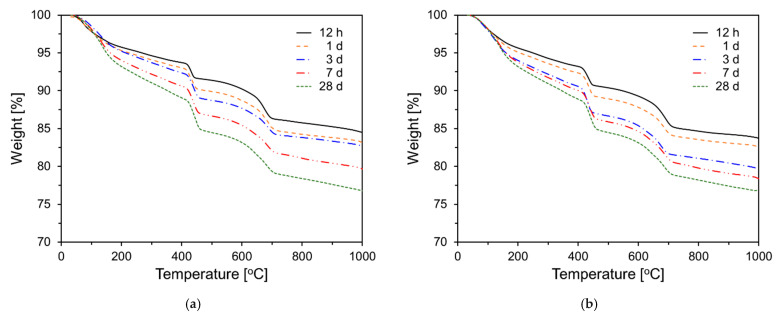

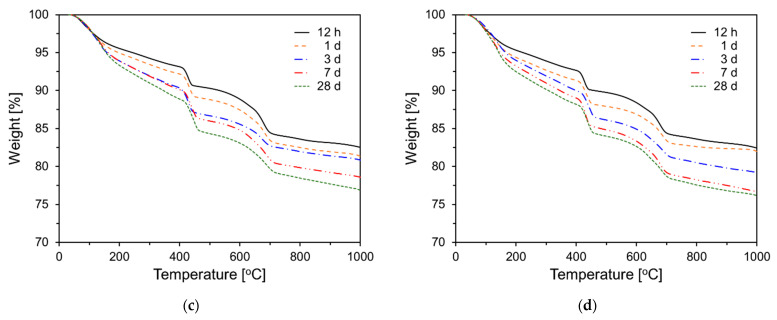
Thermogravimetric analysis results: (**a**) PCSH0; (**b**) PCSH1; (**c**) PCSH2; (**d**) PCSH3.

Figure 11 shows the TG results for PCSH series paste specimens tested at different ages. For all samples, the amount of hydration products tended to increase with increasing age. This is based on the weight changes in the 70–200 °C temperature range, which corresponds to C-S-H and ettringite [48,49]. In addition, this tendency is observed in the 400–500 °C temperature range, which corresponds to Ca(OH)_2_ [50].

**Figure 12:**
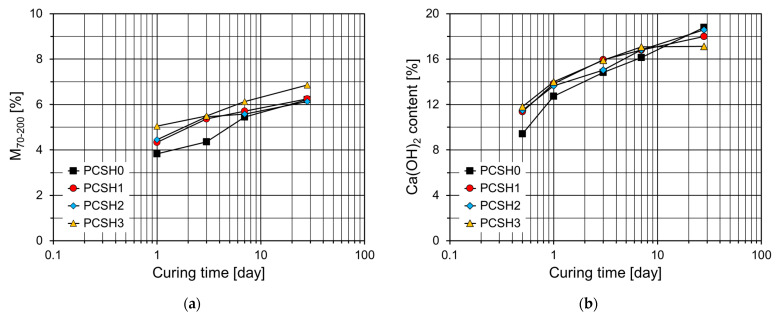
Content of PCSH specimens: (**a**) M70-200; (**b**) Ca(OH)_2_.

As shown in Figure 12a, the mass losses in the 70–200 °C temperature range (M70-200) of specimens containing the C-S-H nanoparticles are higher than those of PCSH0 up to 7 d. This trend is the most noticeable at 3 d, and it increases with an increase in the content of the C-S-H nanoparticles. However, at 28 d, the M70-200 values of PCSH1 and PCSH2 were similar to that of PCSH0. Figure 12b depicts the Ca(OH)_2_ content for each sample at various ages. The largest difference in the Ca(OH)_2_ content was observed at 12 h. Samples containing C-S-H nanoparticles exhibited higher Ca(OH)_2_ contents, with similar trends observed up to 7 d. However, at 28 d, this trend was completely reversed, with the PCSH0 showing the highest Ca(OH)_2_ content and the PCSH3 the lowest. These findings are consistent with the compressive strength results. The compressive strength of PCSH3 exceeded that of PCSH0 for the first three days. However, by seven days, the strength difference had diminished, and at 28 d, PCSH3 exhibited lower compressive strength than PCSH0.

### 3.5. Pore Structure of Specimens Containing C-S-H Nanoparticles

**Figure 13:**
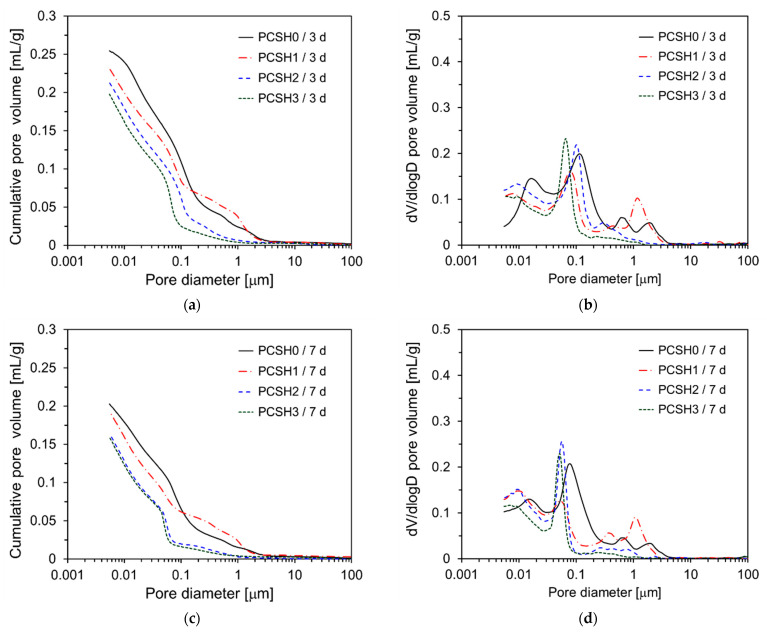

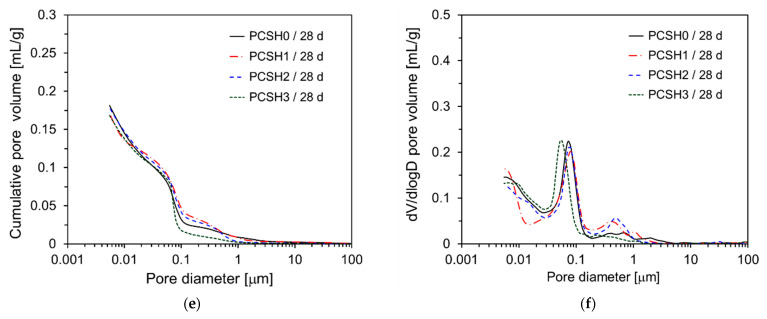
Cumulative pore volume and dV/dlogD curves of specimens: (**a**) Cumulative pore volume at 3 days; (**b**) dV/dlogD curves at 3 days; (**c**) Cumulative pore volume at 7 days; (**d**) dV/dlogD curves at 7 days; (**e**) Cumulative pore volume at 28 days; (**f**) dV/dlogD curves at 28 days.

Figure 13 shows the pore structure of PCSH paste specimens determined by MIP. Figure 13a shows that the cumulative pore volumes of PCSH0, PCSH1, PCSH2, and PCSH3 at 3 d were 0.25, 0.23, 0.21, and 0.20 mL/g, respectively. The unit mL/g represents the volume of mercury intruded per gram of sample, indicating the total pore volume per unit mass of the specimen. The cumulative pore volume exhibits a decreasing trend as the content of C-S-H nanoparticles increases. This is because the microstructure was densified as the C-S-H nanoparticles that act as nucleation sites accelerated the generation of hydration products. A tendency similar to that of 3 d was observed at 7 d, however, the magnitude of the difference decreased. PCSH2 and PCSH3 show very similar values (Figure 13c). All samples exhibit similar values (0.16 to 0.18 mg/L) for the cumulative pore volume at 28 d, similar to the compressive strength tendency shown in Figure 6. The dV/dlogD graphs in Figure 13 show that the pore diameter decreases over time for all samples. Figure 13b shows that the main peak near 1 μm occurs at a smaller pore diameter with an increase in the content of the C-S-H nanoparticles at 3 d; this is attributed to a reduction in the pore size due to the accelerated production of hydration products in the presence of the C-S-H nanoparticles. However, at curing ages of 7 days and 28 days, this trend was not clearly evident. These findings are consistent with the compressive strength results.

## 4. Conclusions

This study investigated the influence of synthesized C–S–H nanoparticles on the early hydration behavior and microstructural development of cement composites. The main findings are summarized as follows:

1. The chemical structure of the synthesized C–S–H nanoparticles, as determined by XPS analysis, was found to be similar to that of 11 Å tobermorite (Ca_4.5_(Si_6_O_16_)(OH)·5H_2_O).
2. From the heat of hydration results, it was confirmed that the C–S–H nanoparticles effectively accelerate early hydration reactions. Isothermal calorimetry revealed that incorporating C–S–H nanoparticles shortened the induction period and increased the intensity of the second exothermic peak.
3. C-S-H nanoparticles enhanced the early-age compressive strength of mortar specimens. However, the 28-day strength slightly decreased due to the formation of a dense hydration product layer in the early stage, which impeded Ca^2+^ diffusion into the un-hydrated cement core, thereby slowing mid-to-late hydration. These trends were consistent with the TG and MIP analyses.
4. C-S-H nanoparticles significantly improved compressive strength by acting as nucleation sites and creating an alkaline environment that promotes the reaction between cement and GGBS.
5. The use of C-S-H nanoparticles as early-strength promoters can support the broader adoption of GGBS in construction materials, thereby contributing to reduced environmental impact from both the cement and steel industries.

## Figures and Tables

**Table 1:** Chemical compositions of raw materials (wt%) [31].

	Chemical Oxide Compositions (wt%)								
	CaO	SiO_2_	Al_2_O_3 _	Fe_2_O_3_	MgO	K_2_O	Na_2_O	SO_3_	LOI
RC	63.7	21.2	4.1	3.6	2.7	1.0	-	1.4	1.3
GGBS	45.7	33.3	13.5	0.8	3.0	0.5	0.3	1.7	1.2

**Table 2:** Mixture proportions of cement composites containing C-S-H nanoparticles.

Specimens	W/B (-)	Binder (kg/m^3^)		C-S-H Nanoparticles (wt%)	Sand (kg/m^3^)	SP (wt%) in the C-S-H Nanoparticle Solution
		RC	GGBS			
PCSH0	0.5	450	-	-	1350	2.4
PCSH1				1.0		
PCSH2				2.0		
PCSH3				3.0		
GCSH0		225	225	-		
GCSH3				3.0		

## Data Availability

The original contributions presented in this study are included in the article. Further inquiries can be directed toward the authors.
